# Analysis of Unconfined Compressive Strength of Rammed Earth Mixes Based on Artificial Neural Network and Statistical Analysis

**DOI:** 10.3390/ma15249029

**Published:** 2022-12-17

**Authors:** Yassir Mubarak Hussein Mustafa, Mohammad Sharif Zami, Omar Saeed Baghabra Al-Amoudi, Mohammed A. Al-Osta, Yakubu Sani Wudil

**Affiliations:** 1 Civil and Environmental Engineering Department, King Fahd University of Petroleum and Minerals, Dhahran 31261, Saudi Arabia; 2 Department of Architecture, Interdisciplinary Research Center for Construction and Building Materials, King Fahd University of Petroleum and Minerals, Dhahran 31261, Saudi Arabia; 3 Civil and Environmental Engineering Department, Interdisciplinary Research Center for Construction and Building Materials, King Fahd University of Petroleum and Minerals, Dhahran 31261, Saudi Arabia; 4 Interdisciplinary Research Center for Construction and Building Materials, King Fahd University of Petroleum and Minerals, Dhahran 31261, Saudi Arabia

**Keywords:** soil stabilization, geotechnical property, cement, neural networks, artificial intelligence, multilinear regression, compressive strength

## Abstract

Earth materials have been used in construction as safe, healthy and environmentally sustainable. It is often challenging to develop an optimum soil mix because of the significant variations in soil properties from one soil to another. The current study analyzed the soil properties, including the grain size distribution, Atterberg limits, compaction characteristics, etc., using multilinear regression (MLR) and artificial neural networks (ANN). Data collected from previous studies (i.e., 488 cases) for stabilized (with either cement or lime) and unstabilized soils were considered and analyzed. Missing data were estimated by correlations reported in previous studies. Then, different ANNs were designed (trained and validated) using Levenberg-Marquardt (L-M) algorithms. Using the MLR, several models were developed to estimate the compressive strength of both unstabilized and stabilized soils with a Pearson Coefficient of Correlation (R^2^) equal to 0.2227 and 0.766, respectively. On the other hand, developed ANNs gave a higher value for R^2^ than MLR (with the highest value achieved at 0.9883). Thereafter, an experimental program was carried out to validate the results achieved in this study. Finally, a sensitivity analysis was carried out using the resulting networks to assess the effect of different soil properties on the unconfined compressive strength (UCS). Moreover, suitable recommendations for earth materials mixes were presented.

## 1. Introduction

It is well recognized that earth constructions are among the oldest types of techniques and earth-manufactured structures are still in use today. The earth structures piqued the interest of both scientists and practitioners because of their superior qualities, such as the enhanced structural capability for load-bearing buildings, thermal comfort, low CO_2_ emission, low embedded energy, etc. Many studies have been conducted to evaluate the use of earth materials as a viable alternative for conventional building materials (blocks, concrete, timber, etc.), either in terms of strength [1,2,3,4], durability [5,6,7], or thermal comfort [8,9] in this regard. Since 1997, according to the online database (Scopus), 1104 papers discussing the rammed earth have been indexed in the database. 1009 of these papers were published in the last decade, thereby showing the importance of such a topic [10].

The earth has proven to be more thermally comfortable than other common construction materials [11]. Furthermore, the durability of earth materials has been dramatically enhanced with the addition of cement or other stabilizers (e.g., lime, fly ash, etc.) [5]. As a result, earth materials can compete with other conventional construction materials since earth can contribute to the creation of an environmentally sustainable structure. One of the significant perception of people [12,13,14] that raises some concerns about the usage of earth materials as an appropriate construction material is its strength. A typical earth compressive strength can be as low as 1.3 MPa, the threshold value assigned by the New Zealand Standard for walls. The exact value can be raised to 2 MPa in the case of vertically loaded bricks and 3.6 MPa for the horizontally loaded ones [15,16]. These values can be increased by stabilization (either mechanical or chemical), reaching up to 12 MPa in some cases [17].

The strength of earth materials is influenced by different factors such as contact-level forces that are developed either due to the capillary actions for unstabilized earth [18] or cementation forces (from stabilization) [19]. Therefore, to achieve the desired strength characteristics, the four main components (i.e., gravel, sand, silts/clays, and water) should be mixed in acceptable proportions to achieve the minimum strength requirement. Based on the literature, there are several soil properties associated with the general behavior of the earth’s materials. These properties include grain size distribution [20], consistency [21,22], and mineral composition [23].

It was reported that a fine content (i.e., silt and clay) of 10–50% and sand/gravel content of 45–75% are considered to be an appropriate proportion for earth construction mix [24]. A plasticity index (PI) of 6% will give sufficient consistency to the soil mixture [21]. However, a higher range (15 to 20%) was suggested by another study [24]. Table 1 summarizes the range of recommended properties taken from selected studies. Such significant variations are expected considering the vast diversity and complexity of soil qualities (ascribed to their different origins), resulting in a high level of uncertainty [25]. Hence, engineers and researchers worldwide tend to carry out extensive experimental programs to develop an ideal earth mix with the help of the previously documented studies.

ANNs are computational models inspired by the structure of neural networks in the brain [28]. Such models perform nonlinear mappings between the input variables and the output and are considered powerful tools that can tackle highly complex challenges such as classification and regression analysis [29]. The first mathematical model describing the neurons was introduced by McCulloch and Pitts [30] to describe the activity of the nerves, and the first artificial neural network was presented by Rosenblatt in 1958 [31]. Since then, the application of ANN has been implemented in many disciplines/fields such as medical organization [32] and medicine [33], management and decision-making [34], materials science [35], etc. In civil engineering, numerous studies were conducted worldwide that have demonstrated that ANNs have a high success rate in data prediction and could be utilized to develop sophisticated and elaborate models that tackle various issues [36,37]. For geotechnical engineering, the application of ANN has been explored in different topics such as landslides’ monitoring and analysis [38,39,40], shear strength analysis [41,42], bearing capacity of footings [43], piles friction ratio [44], and consolidation [45]. Recent thorough reviews have been presented on applying deep learning in geotechnical engineering [46,47].

Compressive strength is the fundamental requirement of earth material performance. Other factors, such as the maximum dry density (MDD) and the optimum moisture content (OMC), are regarded as control variables that are critical in the field preparation of earth mixtures. Therefore, forecasting such influential parameters would be vital. Alavi et al. [48] constructed three different ANNs to predict the compaction properties of stabilized soils: first to predict the OMC, second to predict the MDD, and a third network to predict both the OMC and MDD (i.e., multiple outputs). Their analysis investigated three main stabilizers: Cement, lime, and asphalt. Their input data included the consistency specifications, the sand and clay content, and the stabilizer content. The predictions of the developed ANNs were satisfactory when compared to the experimental results (R^2^ = 0.9896 and 0.9872 for MDD and OMC, respectively). Additionally, it was concluded that the effect of the stabilizer content was negligible and that both MDD and UCS could be predicted using the consistency limits and grain size distribution.

Several studies were carried out to assess the compressive strength of stabilized soils. Das et al. [49] built different ANNs using different loss/cost algorithms, such as Levenberg-Marquardt, to predict the UCS. The models could accurately predict the UCS using base properties such as grain size distribution and consistency limits. Moreover, it was found that implementing the Levenberg-Marquardt (L-M) algorithm produced the best performance compared to other algorithms such as the Bayesian Regularization (BR). Another study was carried out by Mozumder and Laskar [50] to predict the UCS of clay stabilized by geopolymer. Unlike other studies, different inputs were considered along with the consistency limits for this research; these parameters included the percentages of both fly ash and ground-granulated blast-furnace slag, molar concentration, silica to alumina ratio, etc. This is ascribed to the geo-polymerization reactions, which involve a reaction between the alumino-oxide and the alkali polyciliate. Hence, it is expected that such a reaction would be the primary source of the materials’ UCS. With such information, high-accuracy predictions were obtained using the ANN compared to the multivariable regression. Pham et al. (2021) [51] carried out a similar study where 12 inputs were considered, including the grain size distribution, curing period, and the chemical composition of the soil. The developed ANN equation predicted the UCS values based on the inputs with high correlation. Moreover, their results proved the superiority of the ANN over the multivariable regression. One main concern was that all the previous studies were carried out to analyze the behavior of stabilized soils. However, many countries in the world are still using unstabilized soils to prepare earth bricks and use them in various methods of construction [52]. It is important to note that almost 50% of the world population currently lives in earth-based buildings. Therefore, the need to analyse the behaviour of unstabilized soils is essential.

As a result of the preceding details, it is highlighted that similar methodologies can be used to research earth materials. After a thorough review of the literature, it appears that only a few articles have addressed the importance of applying AI concepts to earth material analysis [53,54,55]. Additionally, the effect of applying various types of stabilizers in addition to the cement (in a single network) has not been previously examined. Some characteristics have not been explored in previous AI research, such as the aspect ratio and materials condition during testing (e.g., wet or dry). However, both parameters affect the materials’ performance, as stated in earlier studies [56,57]. Table 2 highlights some of the studies where neural networks were developed to study stabilized soil characteristics. Moreover, the learning/loss algorithms used in these studies and the models’ accuracy are presented.

Based on the discussion carried out in this section, the current study was carried out to find appropriate means in artificial intelligence and statistical analysis concepts. The created tool can be used to predict the performance of earth materials by predicting the unconfined compressive strength of different earth mixes. The developed tool is intended to help minimize the tedious work associated with the soil mix design (for either stabilized or unstabilized soils), and to enhance the soil selection criteria for future studies. The study discusses the application of neural tools in geotechnical engineering and how they can be implemented in earth materials studies. Additionally, multilinear regression analysis (MLR) was applied, and the results of both techniques were compared. The outcome of this study is expected to confirm the use of artificial neural networks in predicting earth materials performance.

## 2. Methods

**Figure 1:**
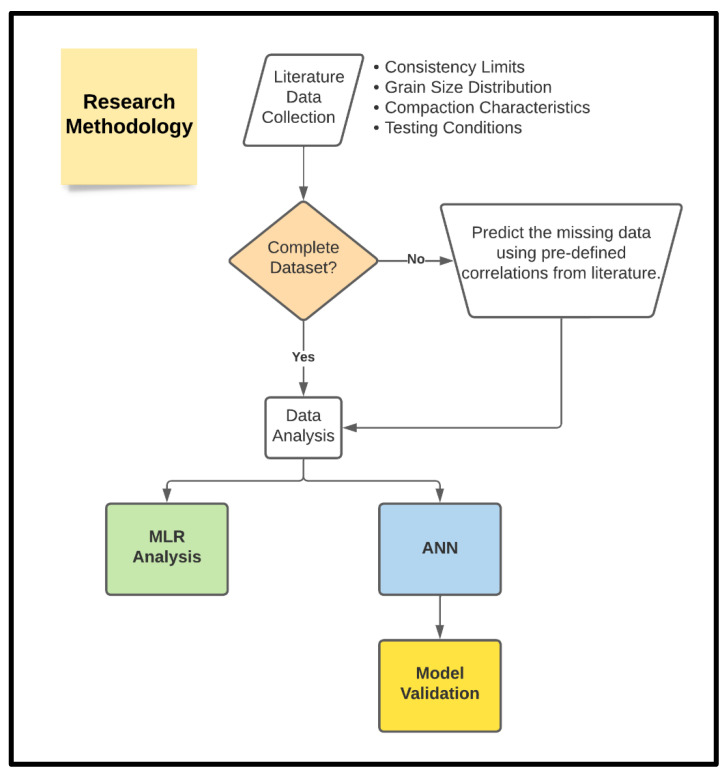
Research methodology.

Several steps were taken to accomplish the aim of this study. The research methodology is depicted in Figure 1. These steps will be addressed in detail in the following Section 2.1–Section 2.5.

### 2.1. Data Collection

Data were collected from previous studies (488 cases) to achieve the goals of this research. According to the literature, numerous factors, such as particle size distribution and consistency, affect earth materials’ performance (i.e., strength in particular). As a result, the following characteristics (inputs) were considered as independent variables that contribute to the 28-day unconfined compressive strength (UCS): Gravel (G); Sand (S); Fines (F); Liquid limit (LL); Plasticity index (PI); Linear shrinkage (LS); Stabilizer type (ST); Stabilizer dose (SD); Optimum moisture content (OMC); Maximum dry density (MDD); Aspect ratio (AR); and Testing condition (DW). To simplify the analysis, the fine content represents both the silt and clay content in the mix. The testing condition (DW) specifies the moisture content of the samples at the time of testing, whether they are dry (i.e., oven-dried) or wet (i.e., without drying), whereas AR specifies the sample size (height/width). Depending on their testing conditions, there is a significant strength variance amongst materials [57,62]. On the other hand, sample sizes and shapes differed among several studies, with some using cylinders [63] whereas others used prisms [64]. As a result, AR was employed as a quantitative variable to describe the sample size in the current investigation. It appeared that neither AR nor DW has been addressed in earlier AI research or included in any existing neural network studies; therefore, they were chosen in this research to study their influence on soil properties.

Mainly, there are two different types of stabilization, either chemical (using additives such as cement, lime, etc.) or physical by changing the soil grain size distribution [65]. For the current study, ST refers to the chemical stabilizer since the mechanical stabilization can be incorporated by changing the proportions of the parameters G, S, and F. Cement and lime are the most frequent stabilizers used to improve soil properties. Both stabilizers contribute to the soil’s strength via the inter-granular bonding produced by the cementitious process (i.e., hydration). Both stabilizers, however, have distinct qualities that make them suited for specific types of soils. Lime, for example, is chosen for treating clay because of its capacity to affect the consistency of the clay [66]. On the other hand, cement is the primary stabilizer appropriate for treating a broader range of soils, including granular and silty soils [67]. As a result, two types of stabilizers were chosen for the current study: Ordinary cement and lime. It must be noted that only the cases of stabilization with either cement or lime were considered for the current study. Cases where combinations of both cement and lime were used would be considered in future studies. Numerical values were assigned to the definite parameters (ST and DW) to simplify the analysis as Table 3 follows:

### 2.2. Data Preparation

Table 4 summarizes the studies from which the data were collected. Specific dimensions of the tested samples (to define the AR) and the number of dosages can be found in detail within the referenced studies. It is noted that finding a single study that contained all the essential data was challenging. Several methods can be utilized to estimate missing data, such as multiple imputations (MI), random forest regression (RF), and support vector regression (SR) [68]. However, after reviewing the data collected from the literature in this study, available correlations from the literature were considered to estimate those missing numbers. Many correlations between different soil characteristics have been developed in the literature. Some of these equations were used in the current study, as shown in Table 5. Those equations were developed based on validated experimental works as presented in previous studies. The equations were used in considering the conditions mentioned in the table to ensure the accuracy of the predictions in the current study.

**Figure 2:**
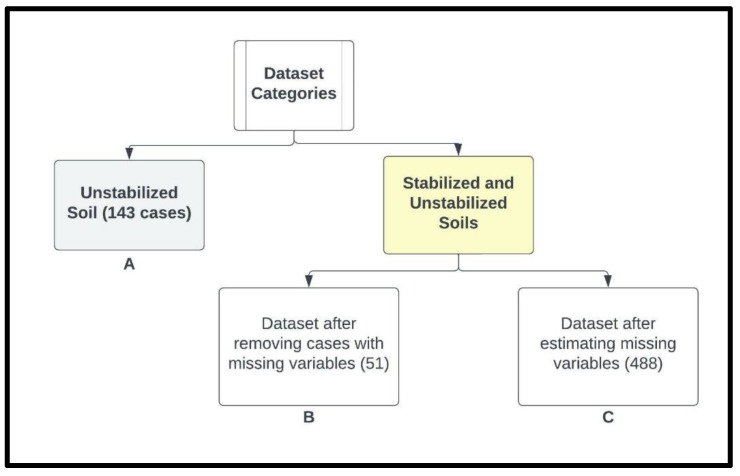
Dataset categories and their associated numbers of data points.

The datasets (i.e., a total of 488 cases) were categorized into three groups, as follows (Figure 2):

- Dataset (A): Only considering the 143 unstabilized cases;
- Dataset (B): All cases with any missing parameters were removed, reducing the dataset size to only 51; and
- Dataset (C): All missing data were estimated using the equations presented in Table 5, and all cases were included (488).

Table 6 displays the data statistics for the input variables. The data were analyzed based on the three scenarios, and the results are compared.

The neural network in the current study was developed using Matlab [103], and the data were scaled/normalized to improve the model’s accuracy due to the considerable variation between the densities (measured in kg/m^3^) and the other input variables [48]. One of the simplest methods for data normalization is min-max normalization, which converts the data’s range to a specific scale ([0, 1] or [−1, 1]). This was accomplished using Equation (1), where the input data were scaled to the range [0, 1]:

(1) $$x_{n}=\;\frac{x-x_{\min}}{x_{\max}-x_{\min}}$$

where x_n_ is the normalized input; x_max_ and x_min_ are the maximum and minimum values of the input, respectively. Using this method helps keeping the relationship between the input data before normalization (especially since the equations in Table 5 were used to estimate missing data) [104]. For output, Equation (2) was used to normalize the data to change the distribution from logarithmic to normal:

(2) $$y_{\log}=\;\ln\left(1+y\right)$$

where y is the output. Moreover, To evaluate the performance of the developed networks, both the root mean square error (RMSE) and Pearson Coefficient of Correlation (R^2^) between the predicted and actual output are calculated as follows:

(3) $$\mathrm{RMSE}=\;\sqrt{\frac{\sum_{n}{\left(y_{m}-y_{p}\right)}^{2}}{n}}$$

(4) $$R^{2}=\;{\left(\frac{n(\sum^{\;}y_{m}y_{p})-(\sum^{\;}y_{m})\left(\sum^{\;}y_{p}\right)}{\sqrt{\left[n\sum^{\;}y_{m}{}^{2}-{\left(\sum^{\;}y_{m}\right)}^{2}][n\sum^{\;}y_{p}{}^{2}-{\left(\sum^{\;}y_{p}\right)}^{2}\right]}}\right)}^{2}$$

where y_m_ is the measured output (from the experiment), y_p_ is the predicted output from the neural network, and *n* is the number of measured data.

### 2.3. Multilinear Regression Analysis (MLR)

One of the simplest forms of correlation between two variables is linear regression. Usually, it is easier to assume that different variables will have some linear correlations. Therefore, along with the ANNs developed in this study, an MLR analysis was carried out, and the results of both approaches were compared based on the developed R^2^ between the predicted and the actual results. Equation (5) shows the general form of MLR [105]:

(5) $$y_{i}=\beta_{0}+\beta_{1}x_{1}+\beta_{2}x_{2}+\ldots +\beta_{n}x_{n}+\epsilon$$

where y_i_ refers to the dependent variable related to the independent variables x_1_, x_2_,…, x_n_, β_0_,…, β_n_ are the parameters for each independent variable, and *ε* is the residual.

Unfortunately, one of the main issues when using MLR is multicollinearity, which occurs when some predictor variables have high inter-correlation [106]. This phenomenon is highly expected in the analysis of soil properties since some parameters can be estimated from gravel and sand content, such as fines content. Hence, only datasets A and B were considered for the MLR analysis. This decision was taken to avoid using scenarios with missing data since they were estimated using different correlations and estimation techniques, as mentioned in Section 2.2. Both datasets A and C were analyzed first without modifications. Then, a stepwise analysis was used to develop the model using the minimum number of variables. A stepwise analysis allows the model to use only those variables that have a significance level (*α*) less than 0.05, and their effect on the outcome is marginal [107]. Finally, the data were refined using the Principal Component Analysis (PCA) to solve the multicollinearity problem, and the analysis was carried out for the third time [106]. All analyses were carried out using IBM statistical software (SPSS Statistics) [108].

### 2.4. ANN Development

A neural network is a complicated structure that involves much learning, differentiating, and optimization processes based on the errors detected. Hence, building a good network requires altering many parameters to achieve the optimum results (i.e., optimization). These parameters include the number of layers, the number of neurons in each layer, activation functions for both hidden layers and output layers, loss function, etc. Other parameters are included for a more comprehensive analysis, such as the network learning rate and its modification during the learning process [37].

In the current study, a comparatively small size of the dataset is considered, and the authors have focused on building simple networks using only one hidden layer (shallow learning). As a result, the parameters considered for optimization were the number of neurons in the hidden layer, the activation function, and the learning rate. For each scenario, the network was built using the loss algorithm Levenberg-Marquardt (L-M). Several studies were conducted using this algorithm, as shown earlier in Table 2. After many trials, it was found that a learning rate of 0.015 would give a sufficient level of accuracy, as shown in Section 3.2. Several trials (five times) were carried out using different numbers of neurons each time to get the optimum number of neurons. Finally, the hyperbolic tangent sigmoid function was used as the transfer function.

Three networks were developed for each dataset (A, B, and C). For each network, the data were divided into training, validation, and testing sets (70, 15, and 15% of the total data, respectively). The data were arranged randomly before splitting them into the sets to prevent the clustering of specific data in one set.

### 2.5. ANN Model Testing

To validate the results of the ANNs prepared in this study, the models were used to estimate the compressive strength of prefabricated stabilized soil samples. The samples were prepared in the lab through an extensive experimental program. Two different types of soils (collected from Riyadh, Saudi Arabia) were stabilized with either cement or lime at different proportions (2.5, 5, 7.5, 10, 12.5, and 15% by the dry weight of the soils).

The stabilized soils were assessed in terms of the properties described in Section 2.1, and the results are shown numerically in Table 7. The consistency limits were measured in accordance with the ASTM standard [109]. Then, the compaction characteristics (i.e., OMC and MDD) were evaluated as per the ASTM standard [110]. After that, all stabilized UCS samples were prepared at their OMC and MDD using specimens with an aspect ratio of 2 (with 36 mm diameter and 72 mm height). The samples were then cured for 28 days (wrapped in plastic sheets to preserve moisture). Finally, they were oven-dried at 60 °C for 48 h to remove the moisture and tested in accordance with the ASTM standard [111].

To analyze the experimental results, two different networks were developed for the different stabilizers (one for cement stabilization (CEM-ANN) and another for lime stabilization (LIM-ANN)) using the data in dataset **C**. For these networks, the data were divided into training and validation sets (70 and 15% of the total data, respectively). Each of the two networks were tested using the experimental UCS results.

## 3. Results and Discussion

### 3.1. MLR Analysis (Dataset A and B)

For unstabilized soil (Dataset A), it was found out that a linear regression between the soil parameters is insignificant and therefore will produce invalid results. Such a conclusion was reached after developing two models; the first one incorporated all parameters (complete model), and the second one did the same after dropping correlated parameters that caused multicollinearity, such as fines content (modified model). However, both models yielded weak correlations (R^2^ = 0.2242), and hence, they were dropped.

**Figure 3:**
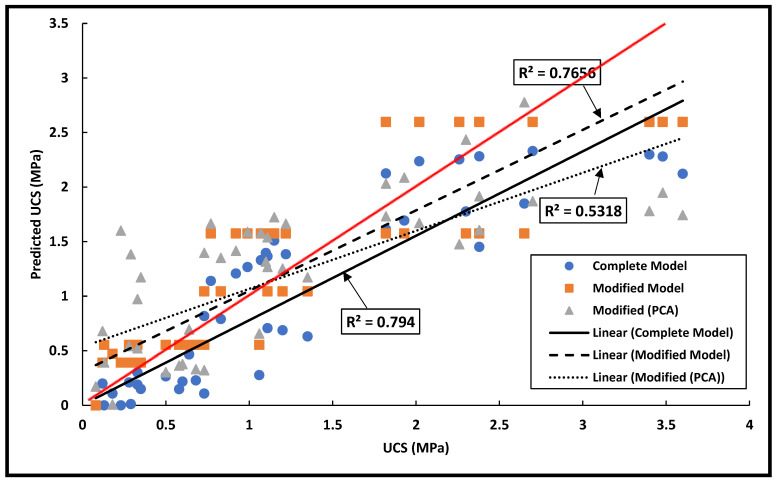
MLR analysis of stabilized soils (Dataset B).

For the stabilized soil (Dataset B), better results were achieved as considering all the input variables compared to unstabilized soils (shown in Figure 3). Hence, the following equation was developed:

(6) $$UCS_{predicted}=2.85-0.047G-0.004S+0.999ST-0.014SD-0.013PI-0.013LS+0.05OMC\;+0.001MDD-2.745AR+0.04DW\;$$

The developed model showed a relatively high level of accuracy with an R^2^ = 0.794. Other studies reported lower values of R^2^ (0.68 and 0.53) [59,61]. The reason for this difference is ascribed to the different input parameters used in these studies, such as the organic matter content.

However, some variables have demonstrated a high level of collinearity when using equation No. 6, such as the grain size distribution parameters (G, S and F). Hence, a stepwise analysis was carried out, and another model was developed with R^2^ = 0.766 and is as follows:

(7) $$UCS_{predicted}=-20.091-0.051G+1.022ST+9.811AR$$

Unfortunately, such a model will not allow an interpretation of the effect of the different variables on the unconfined compressive strength. Moreover, using equation No. 6 would be inconvenient considering the impact of multicollinearity. Hence, another model was built using the PCA. Table 8 shows the different parameters (Comp. 1, 2 and 3) obtained through the PCA and their correlations with the other soil parameters. A regression analysis was carried out using these parameters, and equation No. 8 was developed as follows:

(8) $$UCS_{predicted}=-0.769+0.138\alpha +1.664\gamma +2.255\beta -0.19\delta \;(R^{2}=0.532)$$

where α, γ and β are the normalized coefficients of Comp. 1, 2 and 3, respectively. Though the R^2^ value was marginally smaller than the other two models (Equations (6) and (7)), using this model can help understand the effect of each variable. Figure 3 shows the results for the three developed models and their position from the equity line (R^2^ = 1).

Based on this information, it is noted that using the MLR with stabilized soil was more convenient than with unstabilized soil. A possible reason for this issue is the effect of stabilization since other studies confirmed linear correlations between the stabilizer’s content and the compressive strength of the stabilized soil [56,71,87]. This could increase the linearity of the soil characteristics and enhance the model’s accuracy for the stabilized soil. However, comparing the results with those mentioned in Table 2 shows that, with artificial intelligence, better results with higher R^2^ could be achieved. Therefore, the following section shows the application of ANN on the prepared datasets and the results are compared to those achieved in the case of MLR analyses.

### 3.2. ANN Analysis (All Scenarios)

**Figure 4:**
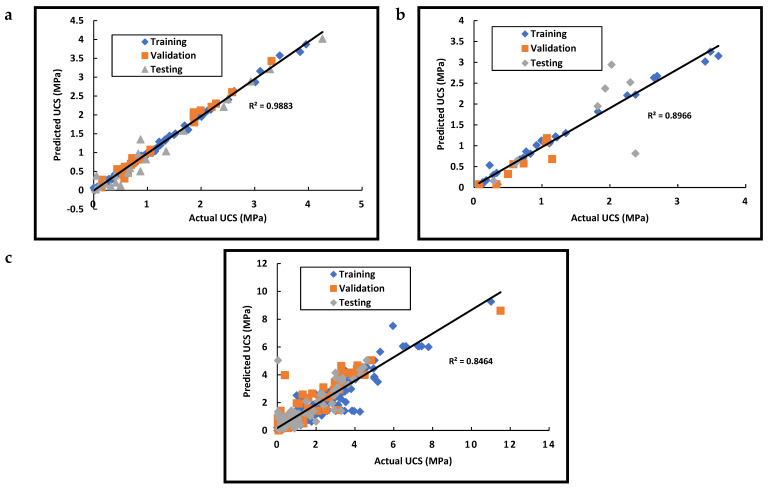
ANN analyses of (**a**) Unstabilized soil (Dataset A); (**b**) Stabilized soil (Dataset B); and (**c**) Stabilized soil (Dataset C).

The numerical indicators for the network performance using the L-M algorithm are shown in Table 9, and the optimum network data are graphically presented in Figure 4. The data presented in Table 9 were gathered from unstabilized soil, a high R^2^ value was obtained in the three cases of training, validation, and testing. An overall R^2^ value of 0.9883 was achieved, as shown in Figure 4a. Such a value was higher than that achieved with the MLR analysis, which proves the ANN was able to catch the performance of unstabilized soil. Moreover, a high R^2^ value in the case of testing proved that such a network could be utilized in predicting unstabilized soil strength for future works. The literature review revealed no studies that assessed the compressive strength of unstabilized soils using machine-learning approaches. Therefore, it can be concluded that the ANN could capture the behavior of unstabilized soil significantly.

For the stabilized soils (both networks B and C), Table 9 shows that the number of neurons needed to achieve the optimum performance had increased significantly as compared to the dataset A network. This increase could be ascribed to the increase in the number of input variables (both ST and SD) and the increase in the complexity of the problem [112].

The two networks (B and C) achieved similar results in R^2^ in terms of the training, validation, and testing phases. However, comparing the errors between both networks, high errors are observed in the case of dataset C. Such a performance could be ascribed to the estimation techniques followed in Section 2.2 (i.e., correlations), which might affect the accuracy of the results. Hence, building bigger datasets in future works with real experimental results would help in utilizing AI in materials mixing design.

As presented in Table 2, several studies were carried out to assess the utilization of AI in stabilized soil assessment. In most of these studies, the models developed incorporated different input parameters and different types of stabilizers [48,113]. Other studies incorporated hybrid stabilization mixtures [114]. Therefore, it can be concluded that the current networks developed in this study will help in understanding the role of different input parameters on the performance of stabilized earth materials. Moreover, incorporating both AR and DW parameters will help in analyzing the effect of the shape and condition of earth structure members on the overall strength.

## 4. ANN Model Testing and Application

**Figure 5:**
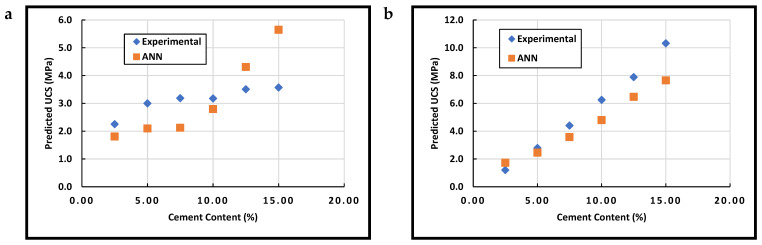
Model Validation for (**a**) Soil-1 and (**b**) Soil-2 (analyzed using the CEM-ANN network).

The experimental results obtained in this study are summarized numerically in Table 10. The results were used to test the networks developed using separated data for both cement and lime stabilization (CEM-ANN and LIM-ANN networks). Figure 5 shows graphically the comparison between the experimental results and those obtained using the CEM-ANN network for soils 1 and 2. For both soils, similar patterns were observed when using the ANN for prediction. Moreover, a strong correlation between the original UCSs and those generated by the neural network was observed in the case of soil 2. For both soils 1 and 2, the ANN was able to simulate the general behavior of cement-stabilized soil where the strength increases as the cement content increases, as seen in previous studies [17,79,81,115,116]. Such behavior is expected considering the hydration of cement, which is the main source of strength in cement-stabilized soil [117].

**Figure 6:**
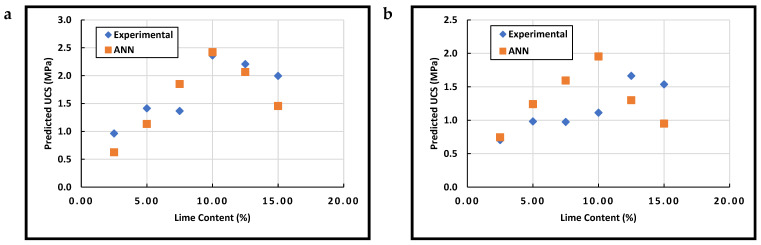
Model Validation for (**a**) Soil-1 and (**b**) Soil-2 (analyzed using the LIM-ANN network).

In the case of lime stabilization, Figure 6 shows marginal differences between the experimental and ANN data obtained using LIM-ANN. A specific lime content is needed to achieve the ultimate strength [17]. In the current study, this content was found to be in the range of 10–12.5%, as shown in Table 10. The developed network (LIM-ANN) was able to predict such content, as shown in Figure 6, which proves the capability of artificial intelligence in the analysis of earth stabilization.

Referring to the data in Figure 5 and Figure 6 (Figure 5a and Figure 6b, specifically), the difference between both the experimental and ANN data could be ascribed to the estimation of the variable by correlations which could affect the accuracy of prediction for the current study data. Moreover, the significant variation in the UCS values obtained in this study covers a wide range of soil properties. Unlike the data obtained from the literature, the two soils presented in the current study had diverse performances in terms of strength. Referring to dataset C (Table 6), there was only one UCS value higher than 10 MPa (i.e., 11.5 MPa achieved at 6% cement stabilization), which was reported by Porter et al. [92]. However, Soil 2 achieved high strengths with cement stabilization at different dosages (which could explain why the network failed to achieve such high strength for Soil-2). Hence, it is expected that some differences in the prediction of the strength would occur due to the insufficient variation in training data.

Based on the previous discussion, it can be concluded that ANNs can be utilized for the future prediction of experimental data. However, the need to develop a large database that could incorporate the varied nature of soils and the significant variations in stabilized soil performance is a must. Doing so will allow future researchers and practitioners to utilize AI in their works, eventually reducing the number of experimental works associated with earth mix design.

## 5. Sensitivity Analysis

After confirming the ability of the networks generated in this study to predict the pattern of experimental results, a sensitivity analysis was carried out to assess the effect of different parameters on the performance of earth materials (in terms of strength) by modifying the soil characteristics. In order to do so, different analyses were carried out by fixing the input variables at their average values (Table 6) and changing the variable under consideration from its minimum to maximum values [118]. The predicted strength was compared to those recommended by different standards (1.3 MPa for rammed walls, 2 and 3.6 MPa for vertically and horizontally loaded bricks, respectively) [15,16]. To perform the analysis, both networks developed in Section 3.2 (Dataset A and C) were used to analyze the unstabilized and stabilized earth performance, respectively.

### 5.1. Unstabilized Earth Materials

Referring to the data used in the current study (Table 6), compressive strength of 4.26 was the highest value found in the literature for unstabilized soils. However, such strength was achieved with AR = 0.5 [98]. In this section, an AR of 2.00 was considered for simplification; hence, we could exclude the usage of these soils in producing horizontally loaded bricks which require a minimum of 3.6 MPa, as stated earlier. Therefore, this section was devoted to analyze the effect of some common soil properties on the compressive strength and to see whether one can achieve the threshold values of 1.3 and 2 MPa.

**Figure 7:**
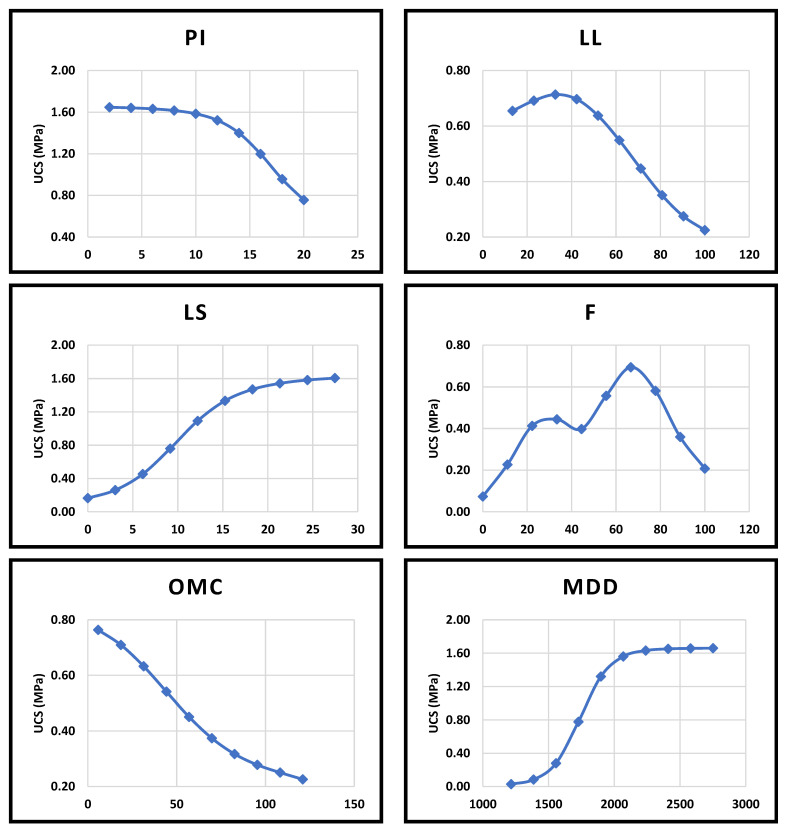
The effect of different parameters on the unconfined compressive strength of unstabilized soil.

Figure 7 shows the effect of different parameters on the strength of unstabilized soils. For the plasticity index, Figure 7 shows that a PI of less than 10% would be sufficient to give the optimum strength for unstabilized soil. Moreover, the analysis concluded that a PI of less than 15% will result in a strength of 1.3 MPa, which is sufficient for a rammed earth wall. Such a result comes in agreement with Burroughs’s recommendation of a maximum PI of 15% for rammed earth applications [22]. Also, Figure 7 shows that the increase in both LL and PI would reduce the compressive strength of the soil, and this agrees with previous studies [91,119]. In addition to both LL and PI, increasing the LS would increase the strength up to a certain limit. It is noted that such behavior was achieved when both PI and LL were fixed at their average values (Table 6), which is not the actual condition. Therefore, such behavior could be assumed to be true only in the case of a constant plasticity index.

For the fines content, Figure 7 shows that an optimum fines content in the range of 30–70% would achieve the optimum strength, which is consistent with Alley’s recommendations [21]. However, a notable breakdown in the tendency of the strength was observed at F ≈ 40% which could be ascribed to the effect of the combination of other parameters that are fixed at their average values as stated earlier. Other explanation of this phenomenon could be the fines content range that was used in training the ANN. Limited data range could affect the accuracy of the prediction in ANN considering the incapability of neural networks in extrapolation [120].

Moreover, as shown in Figure 7, increasing the optimum moisture content would decrease the ultimate strength of the unstabilized soil, and this agrees with previous studies. This is expected since higher moisture content means higher plasticity [25,121].

From the literature, it was found that soil density is the main parameter affecting the strength of unstabilized earth [122,123]. Therefore, the effect of MDD on the compressive strength of unstabilized earth was presented in Figure 7. It was found that a minimum density of nearly 1800 kg/m^3^ is needed to achieve a compressive strength of 1.3 MPa. Such a density could be reduced when stabilizing the soil. Therefore, a common practice would be to find the ultimate combination of both the soil density and stabilizer dosage to achieve the optimum strength [124].

Based on the above information, it can be concluded that one can utilize the advanced tools of AI in optimizing the components of earth mixes to develop stronger mixtures of unstabilized soils. Such mixtures will and still be inconvenient if the aim is to develop strong and durable earth construction materials. Hence, stabilization would still be preferred to achieve the needed requirements.

### 5.2. Stabilized Earth Materials

Similar to the previous section, the current study considered the effect of stabilizer types and dosage on the compressive strength of the soil for the stabilized soil. All other parameters were kept at their average value, as reported in Table 6.

**Figure 8:**
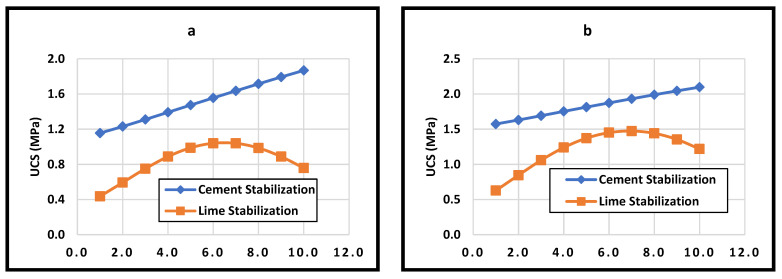

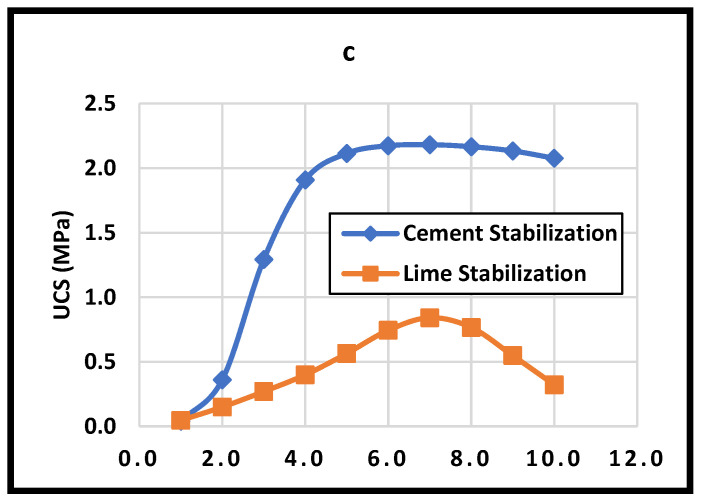
The effect of different parameters on the unconfined compressive strength of stabilized soil. (**a**) All inputs are kept at their average values; (**b**) Soil performance at a density of 1800 kg/m^3^ and 18% OMC; (**c**) Soil performance with the inputs vary as the stabilizer content increase.

Figure 8 shows the compressive strength of both cement and lime-stabilized soil whereby the increase of cement content increased the compressive strength, and this is found in other studies and analysis presented in Section 4 [17,79,81,115,116]. Moreover, the effect of lime stabilization is similar to the experimental work presented in this study and other previous studies [17]. Figure 8 shows that a lime content of nearly 6% would give the ultimate strength of the soil. However, the data in Figure 8a show weak performance in both cement and lime stabilization, which is logical considering that all input variables were kept in their average values. Therefore, the dry density was increased to 1800 kg/m^3^ while keeping the moisture content at 18%. Such practice is expected in the field in order to control the soil mix design. Doing so resulted in increasing the ultimate strength of both cement and lime-stabilized soil, as shown in Figure 8b.

It is commonly known that adding different stabilizers to the soil would change the other attributes, such as the consistency limits and compaction characteristics. A third scenario was considered here by changing the attributes systematically by increasing the stabilizer’s content. As a result, the ultimate strength of the lime-stabilized soil has shifted to the right as it decreased to 0.84 MPa. Moreover, the behavior of the cement-stabilized soil has become nonlinear with an ultimate strength of more than 2 MPa, as shown in Figure 8c.

Based on the above discussion, it can be concluded that using the ANN could enhance the prediction of materials’ behavior and soil mix design for different rammed earth applications. In order to use the ANN tool to predict the behavior of earth materials, we have to recognize the following points:

- ANNs can be fed by wide ranges of data and could predict the results to a high level of accuracy, as stated in Section 3.2. One major issue with the ANN is the lack of extrapolation and hence, it cannot predict values that fall beyond the limits of the data used for the network development [125].
- The predicted results might be unreasonable. This depends entirely on whether the assumed characteristics are logical or not. For instance, increasing the stabilizer content would generally reduce the plasticity index of the soil [81]. Hence, it is usually preferred to take such behavior into account.

## 6. Conclusions

The current study was carried out to study the usage of an Artificial Neural Network as an appropriate tool in analyzing the compressive strength of earth materials and the effect of different parameters such as the soil consistency and compaction characteristics on the material performance. Both ANN and MLR models were developed using three different datasets (for unstabilized and stabilized soils). Twelve inputs were considered for the analysis to predict the unconfined compressive strength and the relevant data were collected from the literature. Missing data were estimated using validated correlations from previous studies. Then, three separate datasets were prepared: Dataset (A) contained all data related to unstabilized soils; Dataset (B), including the stabilized/unstabilized cases after excluding the studies with missing variables; and Dataset (C) had all the data collected from the literature and those estimated by correlations.

The results obtained for the MLR revealed a weak correlation between the unconfined compressive strength and the different input parameters due to several reasons, such as the multicollinearity between the soil characteristics. In the case of ANN, using L-M learning algorithms, several networks (for each dataset) were developed, and their performances were compared in terms of both RMSE and R^2^. For the unstabilized soil, the network developed from dataset A achieved the highest R^2^ (0.9883). On the other hand, for the stabilized soil case, the network developed for dataset B gave an R^2^ value of 0.8966, which is less than that obtained for the unstabilized soil. This result was expected considering the small dataset size for B (51) as compared to the unstabilized ones (143). For dataset C, the Pearson coefficient was decreased to 0.8464, which could be ascribed to the accuracy of estimation methods (Table 5).

To test the accuracy of the ANN, two different networks were developed using the data of both cement and lime stabilization separately (CEM-ANN and LIM-ANN networks, respectively). A thorough experimental program was carried out on two different types of soil collected from Riyadh, Saudi Arabia. The program was carried out to assess the unconfined compressive strength of the soils after stabilizing them with either cement or lime at different percentages (2.5, 5, 7.5, 10, 12.5 and 15% by the dry weight of the soil). The developed networks (CEM-ANN and LIM-ANN) were used to predict the UCS of the experimental data. The networks showed a high capability of predicting the experimental results and the behavior of the stabilized soil. After the model validation, a sensitivity analysis was carried out using the networks developed for datasets A and C. These datasets studied the effect of soil parameters on the strength of the unstabilized/stabilized soils. The results were compared to those proposed by the different rammed earth standards. It was found that a fine content of between 30 and 70% can be used to produce rammed earth walls at specific plasticity conditions and densities. On the other hand, a similar study was carried out on the stabilized soil. The obtained results showed the ANN’s advantage in predicting the stabilized soil’s performance.

The current study serves as guidance in utilizing AI techniques to assess rammed earth structures and soil-stabilizer mix design optimization. Moreover, the suggested technique can optimize other parameters such as gravel, moisture content, aspect ratio, and testing conditions. It is expected that appropriate classification systems could be built for future usage to enhance the quality of earth material studies by using such an advanced tool. Several other parameters, for example, clay mineralogy and other stabilizers such as fly ash, cement kiln dust, rice husk, straw, etc., could be incorporated into such tools. Such a study can be applied using other artificial intelligence techniques such as the support vector regression (SVR) and Decision Trees (DT). Finally, a proper classification system that describes the types of soil and their properties could be built using these tools. A system of this type can be used to estimate whether specific soils are suitable to be used as construction materials.

## Figures and Tables

**Table 1:** Basic properties of soil and their ranges for usage in earth construction.

Study	Gravels and Sand		Silt and Clay		PI		LS	
	Min	Max	Min	Max	Min	Max	Min	Max
[21]	10	20	75	100	NA	6	NA	NA
[26]	65	70	30	35	NA	NA	NA	NA
[24]	45	75	10	50	15	20	NA	NA
[27]	45	75	25	55	NA	NA	NA	NA
[22]	NA	64	21	35	NA	15	NA	6

**Table 2:** Summary of some neural network studies focusing on soil characteristics and strength (Compiled by the authors).

Study	Targeted Outcome	Available Data	ANN Loss Algorithm	Tested Data	R^2^	Most Important Parameters
[58]	Grain size distribution	Cone resistance, sleeve friction, and grain size distribution.	General regression neural network (GRNN)	142	86% success rate	NA
[48]	OMC and MDD	Grain size distribution; Atterberg limits; linear shrinkage (LS); cement, lime, and asphalt content.	Gradient Descent with Momentum (SGDM)	192	0.990 for MDD 0.987 for OMC	Grain size and consistency
[49]	MDD and UCS	Grain size distribution; Atterberg limits; water content; cement content.	Bayesian Regularization; Levenberg-Marquardt; Differential Evolution.	55	0.828	Moisture content and clay content
[50]	UCS	Consistency limits; sand content; fly ash content; ground-granulated blast-furnace slag content; mineralogical compositions.	Bayesian Regularization	283	0.964	Sand and fly ash content
[59]	MDD and UCS	Grain size distribution; Atterberg limits; water content; cement content.	Functional Networks (FN)	55	0.828 for MDD 0.865 for UCS	Moisture and gravels contents
[53]	Soil classification (based on the Unified Soil Classification System)	Quantitative field tests (pen, stick, and shine test); fines content	Scaled Conjugate Gradient	33	89.35% success rate	NA
[60]	UCS and CBR	Micro-silica and lime content; curing period and condition (soaked and unsoaked)	NA	90	0.992 for UCS 0.990 for CBR	Lime and micro-silica contents
[54]	UCS	Grain size distribution; cement content; density; moisture content.	NA	373	0.943	NA
[61]	UCS	Grain size distribution; organic matter; water, cement content, and curing period.	NA	444	0.941	Water/cement ratio, cement content, and organic matter
[55]	UCS; plasticity coefficient; drying shrinkage; shaping moist.	Mineralogical components (macro-oxides, clay contents, etc.)	Broyden-Fletcher-Goldfarb-Shanno (BFGS)	139	0.785	NA
[51]	UCS	Grain size distribution; soil chemical composition; cement content; curing period.	Levenberg-Marquardt	80	0.994	Cement content and soil smaller than 0.5 mm

**Table 3:** Numerical values associated with DW and ST.

**DW**	Wet:	1
	Dry:	2
**ST**	Unstabilized:	1
	Lime stabilized:	2
	Cement stabilized:	3

**Table 4:** Summary of the data sources.

Study	Stabilizer Type	Dosages Range (%)	Soil Conditions
[69]	Lime	0–10	Moderate to highly plastic clay (five types)
[70]	Lime	0–12	Moderate to highly plastic clay (five types)
[71]	Cement and lime	0–9	Highly plastic clay (CH)
[72]	Lime	0–9	Clayey sands
[73]	Cement and lime	0–7	Highly plastic clay
[74]	Cement	0–10	Silty clay (highly plastic)
[75]	Lime	0–8	Highly plastic clay (two types)
[76]	Lime	0–15	Highly plastic (expansive) clay
[77]	Cement	0–12	Four types of laterite soils with different plasticity ranges
[78]	Cement	0–10	Sabkha soil (low plasticity)
[79]	Cement	4–10	Highly plastic clay (three types)
[66]	Lime	0–13	Highly plastic clay
[80]	Lime	0 & 5	Highly plastic clay
[81]	Cement and lime	0–8	Organic clay with different plasticity ranges (eight types)
[82]	Unstabilized	0	Low plastic residual soils (four types)
[83]	Cement	0–5	Several types of soil with different clay contents
[84]	Lime	0–8	Highly plastic (expansive) clay
[85]	Cement	5–15	Highly plastic clay (Kaolinite)
[62]	Lime	0–6	Several types of soil with different clay contents
[86]	Lime	0–6	Highly plastic clay
[56]	Cement	10	Clayey sand
[87]	Cement	0–7.5	Sandy soil
[88]	Cement and lime	0–9	Low plasticity clay
[89]	Cement	0–15	Highly plastic clay (CH)
[90]	Cement	8–10	Clayey sand (high plasticity)
[91]	Unstabilized	0	Five types of Kaolinite clays (low plasticity)
[68]	Lime	0–10	Highly plastic clay (lateritic soil)
[64]	Cement	7–10	Clayey sand
[17]	Cement and Lime	0–9	Clayey sand with expansive nature and high plasticity
[92]	Cement	0–6	Clayey sand with gravel (low plasticity)
[93]	Cement	0–12	Low plasticity organic clay (CL)
[94]	Lime	0–12	Highly plastic clay (CH)
[95]	Cement	3–7	Highly plastic clay (Kaolinite)
[57]	Cement and lime	5–15	Several types of low clay contents and medium plasticity
[96]	Cement	0–12	Several types of soil with different plasticity
[97]	Cement	0–12	Highly plastic clay (lateritic soil)
[98]	Unstabilized	0	Four types of medium to high plasticity

**Table 5:** Some correlations used for soil characteristics estimation.

	Missing Data	Available Data	Suggested Correlation	Condition	Reference
**1**	OMC and MDD	Consistency limits (LL, PI	OMC=−0.86LL+3.04(LLGs)+2.2	10 < FC < 100 MDD > 2038.74 kg/m^3^ OMC < 10%	[99]
			$\mathrm{MDD}=\left(\left(40.316\times {\mathrm{OMC}}^{-0.295}\right)\times {\mathrm{PI}}^{0.032}\right)-2.4$		
**2**	LS	PI	LS=PI2.13		[100]
**3**	OMC and MDD	LL, PL	*OMC* = 0.94*PL*	41 < FC < 99	[101]
			*OMC* = 0.52*LL*		
			*MDD* = 0.22 (96.32 − *PL*)		
			*MDD* = 0.09 (225.78 − *LL*)		
**4**	LL and PI	FC	*LL* = 0.67*FC*	Better used for sand-Kaolinite mixtures	[102]
			*PI* = 0.96*LL* − (0.26*FC* + 10)		

**Table 6:** Descriptive statistics of the input variables.

	Gravel (%)	Sand (%)	Fines (%)	LL (%)	PI (%)	LS (%)	OMC (%)	MDD (kg/m^3^)	SD (%)	UCS (Mpa)
**Unstabilized Soil (Dataset A)**										
**Min**	0	0	0	13.40	1.00	0.00	5.80	1216.00	-	0.00
**Max**	62.00	100.00	100.00	100.00	58.00	27.23	121.00	2750.00	-	4.26
**Average**	4.09	29.92	65.99	43.28	20.87	8.55	20.56	1705.57	-	0.98
**Std. Deviation**	12.32	26.29	30.95	15.19	11.06	6.31	10.56	257.88	-	0.92
**Stabilized Soil (Dataset B)**										
**Min**	0	0	12.30	26.30	3.00	1.00	13.50	1068.00	0	0.08
**Max**	23.20	76.81	100.00	100.00	47.00	20.00	33.60	1779.00	15.00	3.60
**Average**	2.76	48.35	48.89	42.46	18.47	7.45	20.85	1479.46	3.27	1.14
**Std. Deviation**	7.47	26.70	29.24	13.46	10.02	5.26	4.95	158.37	3.80	0.91
**Stabilized Soil (Dataset C)**										
**Min**	0	0.00	0.00	3.35	1.00	0.00	4.68	641.09	0.00	0.00
**Max**	62.00	100.00	100.00	100.00	63.00	29.58	121.00	2750.00	15.00	11.50
**Average**	4.51	36.04	59.44	40.36	17.76	6.26	22.04	1672.53	5.16	1.69
**Std. Deviation**	11.49	29.76	33.17	17.36	10.91	6.03	12.91	280.84	4.27	1.58

**Table 7:** Geotechnical properties of the selected soils.

Test	Soil 1	Soil 2
**Specific gravity**	2.50	2.45
**LL (%)**	33.49	17.52
**PI (%)**	17.78	2.28
**Grain Size Distribution**		
G (%)	0	0
S (%)	84.7	74
F (%)	15.3	26
**MDD (kg/m^3^)**	1730.89	1800.20
**OMC (%)**	19.2	13.48
**UCS (Mpa)**	2.12	0.62

**Table 8:** PCA developed components.

	Comp. 1	Comp. 2	Comp. 3	Comp. 4
**G**	−0.083	0.063	−0.082	0.285
**S**	−0.204	0.123	−0.195	−0.058
**F**	0.207	−0.129	0.199	−0.020
**ST**	−0.044	0.261	0.217	0.116
**SD**	0.026	0.272	0.272	0.138
**LL**	0.206	0.122	−0.126	0.026
**PI**	0.205	0.016	−0.207	−0.004
**LS**	0.142	−0.050	−0.346	0.180
**OMC**	0.151	0.174	0.027	−0.394
**MDD**	−0.051	−0.297	0.100	0.362
**AR**	0.015	−0.232	0.224	−0.409
**DW**	0.160	0.014	0.188	0.446

**Table 9:** ANNs performance comparison for the different datasets at training/validation/testing stages.

Dataset	No. of Neurons	Epoch	Training		Validation		Testing		Overall R^2^
			R^2^	RMSE	R^2^	RMSE	R^2^	RMSE	
A	24	14	0.9976	0.0392	0.9868	0.1984	0.9698	0.168	0.9883
B	41	9	0.9877	0.1345	0.8012	0.2096	0.5555	0.6673	0.8966
C	44	17	0.8909	0.5478	0.8304	0.7492	0.6622	0.8024	0.8464

**Table 10:** Experimental results for model validation.

Study	Soil Gradation %			SD (%)	Consistency (%)			Compaction		AR	D/W	UCS (MPa)
	G	S	F		LL	PI	SL	OMC	MDD			
**Soil-1 (Cement)**	0.00	84.70	15.30	2.50	29.83	15.55	7.30	18.24	1733.10	2.00	2.00	2.251
	0.00	84.70	15.30	5.00	29.78	17.72	8.32	18.80	1736.85	2.00	2.00	2.999
	0.00	84.70	15.30	7.50	26.96	3.19	1.50	19.36	1740.60	2.00	2.00	3.185
	0.00	84.70	15.30	10.00	19.80	3.35	1.57	19.92	1744.34	2.00	2.00	3.179
	0.00	84.70	15.30	12.50	18.74	2.12	1.00	20.48	1748.09	2.00	2.00	3.511
	0.00	84.70	15.30	15.00	18.64	2.79	1.31	21.04	1751.83	2.00	2.00	3.570
**Soil-1 (Lime)**	0.00	84.70	15.30	2.50	29.83	17.9	8.40	19.78	1703.70	2.00	2.00	0.963
	0.00	84.70	15.30	5.00	29.84	13.90	6.53	20.85	1679.15	2.00	2.00	1.415
	0.00	84.70	15.30	7.50	30.39	4.96	2.33	21.93	1654.61	2.00	2.00	1.367
	0.00	84.70	15.30	10.00	32.89	5.49	2.58	23.00	1630.07	2.00	2.00	2.364
	0.00	84.70	15.30	12.50	32.93	3.98	1.87	24.07	1605.53	2.00	2.00	2.209
	0.00	84.70	15.30	15.00	32.94	1.00	0.47	25.14	1580.99	2.00	2.00	1.997
**Soil-2 (Cement)**	0.00	74.00	26.00	2.50	29.83	4.41	2.07	14.85	1772.60	2.00	2.00	1.200
	0.00	74.00	26.00	5.00	29.80	4.39	2.06	15.25	1778.64	2.00	2.00	2.784
	0.00	74.00	26.00	7.50	27.56	5.14	2.41	15.65	1784.68	2.00	2.00	4.408
	0.00	74.00	26.00	10.00	19.81	3.96	1.86	16.04	1790.72	2.00	2.00	6.254
	0.00	74.00	26.00	12.50	18.70	2.98	1.40	16.44	1796.76	2.00	2.00	7.892
	0.00	74.00	26.00	15.00	18.62	0.91	0.43	16.83	1802.80	2.00	2.00	10.320
**Soil-2 (Lime)**	0.00	74.00	26.00	2.50	29.83	3.84	1.80	15.55	1769.70	2.00	2.00	0.704
	0.00	74.00	26.00	5.00	29.83	3.41	1.60	16.49	1748.78	2.00	2.00	0.983
	0.00	74.00	26.00	7.50	29.82	5.65	2.65	17.43	1727.85	2.00	2.00	0.975
	0.00	74.00	26.00	10.00	29.52	2.63	1.23	18.38	1706.93	2.00	2.00	1.114
	0.00	74.00	26.00	12.50	28.11	4.85	2.28	19.32	1686.01	2.00	2.00	1.665
	0.00	74.00	26.00	15.00	27.82	2	0.94	20.26	1665.09	2.00	2.00	1.536

## Data Availability

The data used in the current study are collected and compiled by the authors using the references presented in Table 4. The data presented in this study are available on request from the corresponding author.
